# Prediction of lung cancer risk in Chinese population with genetic‐environment factor using extreme gradient boosting

**DOI:** 10.1002/cam4.4800

**Published:** 2022-05-02

**Authors:** Yutao Li, Zixiu Zou, Zhunyi Gao, Yi Wang, Man Xiao, Chang Xu, Gengxi Jiang, Haijian Wang, Li Jin, Jiucun Wang, Huai Zhou Wang, Shicheng Guo, Junjie Wu

**Affiliations:** ^1^ School of Life Sciences Fudan University Shanghai China; ^2^ Company 6 of Basic Medical School Navy Military Medical University Shanghai China; ^3^ Department of Biochemistry and Molecular Biology Hainan Medical University Haikou China; ^4^ Clinical College of Xiangnan University Chenzhou China; ^5^ Department of Thoracic Surgery the First Affiliated Hospital of Naval Medical University (Second Military Medical University) Shanghai China; ^6^ Department of Laboratory Diagnosis the First Affiliated Hospital of Naval Medical University (Second Military Medical University) Shanghai China; ^7^ Department of Pulmonary and Critical Care Medicine, Zhongshan Hospital Fudan University Shanghai China; ^8^ Department of Pulmonary and Critical Care Medicine Shanghai Geriatric Medical Center Shanghai China

**Keywords:** Chinese population, extreme gradient boosting, lung cancer, risk model, single nucleotide polymorphisms

## Abstract

**Background:**

Detecting early‐stage lung cancer is critical to reduce the lung cancer mortality rate; however, existing models based on germline variants perform poorly, and new models are needed. This study aimed to use extreme gradient boosting to develop a predictive model for the early diagnosis of lung cancer in a multicenter case–control study.

**Materials and Methods:**

A total of 974 cases and 1005 controls in Shanghai and Taizhou were recruited, and 61 single nucleotide polymorphisms (SNPs) were genotyped. Multivariate logistic regression was used to calculate the association between signal SNPs and lung cancer risk. Logistic regression (LR) and extreme gradient boosting (XGBoost) algorithms, a large‐scale machine learning algorithm, were adopted to build the lung cancer risk model. In both models, 10‐fold cross‐validation was performed, and model predictive performance was evaluated by the area under the curve (AUC).

**Results:**

After FDR adjustment, TYMS rs3819102 and BAG6 rs1077393 were significantly associated with lung cancer risk (*p* < 0.05). For lung cancer risk prediction, the model predicted only with epidemiology attained an AUC of 0.703 for LR and 0.744 for XGBoost. Compared with the LR model predicted only with epidemiology, further adding SNPs and applying XGBoost increased the AUC to 0.759 (*p* < 0.001) in the XGBoost model. BAG6 rs1077393 was the most important predictor among all SNPs in the lung cancer prediction XGBoost model, followed by TERT rs2735845 and CAMKK1 rs7214723. Further stratification in lung adenocarcinoma (ADC) showed a significantly elevated performance from 0.639 to 0.699 (*p* = 0.009) when applying XGBoost and adding SNPs to the model, while the best model for lung squamous cell carcinoma (SCC) prediction was the LR model predicted with epidemiology and SNPs (AUC = 0.833), compared with the XGBoost model (AUC = 0.816).

**Conclusion:**

Our lung cancer risk prediction models in the Chinese population have a strong predictive ability, especially for SCC. Adding SNPs and applying the XGBoost algorithm to the epidemiologic‐based logistic regression risk prediction model significantly improves model performance.

## Abstract 2

We use Extreme Gradient Boosting to build a lung cancer prediction model with AUC = 0.759. In this model, BAG6 rs1077393 was the most important predictor. Compared with logistic regression model, applying XGBoost algorithm significantly improves the model performance.

## 1 INTRODUCTION

Lung cancer is the most common cause of cancer mortality, and its 5‐year survival rate is less than 20% overall and 56% in the early stages.^1^ Early diagnosis and interventions for lung cancer are crucial for the extension of survival time. One of the early detection methods is low‐dose computed tomography (LDCT), but low population coverage and X‐ray exposure limit its application in lung cancer screening.^2^, ^3^, ^4^ SNP‐based risk prediction models are appropriate tools for preventive interventions that provide an estimate of the risk of developing lung cancer.^5^ A total of 301 SNPs combined with smoking pack‐years have a model prediction performance area under the curve (AUC) value of 0.656 in Caucasians.^6^ The smoking duration (years)‐based model has an AUC of 0.75, and 20 further combined SNPs resulted in an AUC up to 0.81 in Caucasians.^5^

Logistic regression is the most widely used method for model building because of its efficiency and interpretability. However, there are several disadvantages in the application of logistic regression. First, the assumption of linearity in logistic regression is rarely established. Next, the use of the coefficient present in logistic regression as an odds ratio does not consider the association with other independent variables. Moreover, logistic regression is not sufficiently robust when a strongly influential outlier is present.^7^ The extreme gradient boosting (XGBoost) algorithm, a large‐scale machine learning algorithm, is an efficient and scalable variant of gradient boosting and can be used for both classification and regression problems. XGBoost has resulted in the best AUC compared to other machine learning algorithms for predicting the stage of cancer patients.^8^

To our acknowledge, several studies have included SNPs in model‐building in the Chinese population.^9^, ^10^, ^11^ All of them used logistic regression, and none of these models had sufficient predictive performance, with an AUC <0.7. In this study, we constructed susceptibility models using epidemiologic information and 61 SNPs by using logistic regression and machine learning methods for lung cancer and stratifications such as lung adenocarcinoma (ADC) and lung squamous cell carcinoma (SCC). The area under the receiver operating characteristic (AUC) was used to assess the contribution of the presence of SNPs and machine learning methods in the risk prediction models.

## 2 MATERIALS AND METHODS

### 2.1 Study subjects

We recruited 974 cases and 1005 controls for the case–control study of the Chinese Han population. Briefly, lung cancer patients diagnosed between March 2005 and January 2010 were recruited from Shanghai and Taizhou, as shown in Table S1. The inclusion criteria for patients were histologically or cytologically confirmed ADC, SCC, adenosquamous, or small cell lung cancer. Control subjects with no individual history of any cancer were recruited, and case subjects were matched by age and sex. Every subject was interviewed to collect epidemiological and clinical data including sex, age, smoking intensity, smoking duration, family history of lung cancer (family history), lung cancer histology, and lung cancer stage. All participants signed informed consent, and the study was approved by the ethics committee of the School of Life Sciences, Fudan University.

### 2.2 SNP selection and genotype detection

**FIGURE 1:**
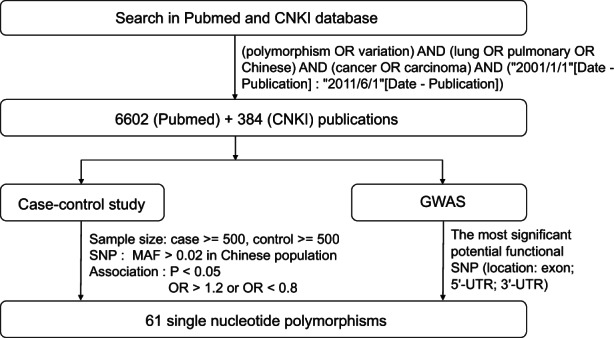
Selection criteria of single nucleotide polymorphisms. CNKI, China National Knowledge Infrastructure; GWAS, Genome Wide Association Study; SNP, single nucleotide polymorphisms; MAF, minor allele frequency; OR, odds ratio

Approximately 3–5 ml of peripheral blood was collected from each individual. Genomic DNA was extracted from whole blood samples by using the Qiagen Blood Kit (Qiagen) following the manufacturer's instructions. SNPs were selected from among those that were significantly associated with lung cancer in genome‐wide association studies (GWAS)^12^, ^13^, ^14^ or association studies,^15^, ^16^, ^17^, ^18^, ^19^, ^20^, ^21^ as shown in Figure 1 and Table S2. Genotyping was performed as previously described.^22^ Successful genotyped SNP criteria were as follows: call rate >95%, control group Hardy–Weinberg equilibrium (HWE) *p* > 0.01, and minor allele frequency (MAF) >0.01.

### 2.3 Statistical analysis

Pearson's chi‐squared test or Student's *t*‐test was used to evaluate clinical and environmental factor differences between the case and control groups. Univariate unconditional logistic regression was used to estimate the association between SNPs and the risk of lung cancer. The odds ratio (OR) and 95% confidence interval (CI) were calculated by adjusting for age and gender in the additive, dominant, and recessive models of SNPs. *p*‐values were adjusted for multiple testing by using the false discovery rate (FDR) method.

Two types of models were built using 61 SNPs and age, gender, smoking intensity, smoking duration, and family history: logistic regression (LR) and extreme gradient boosting (XGBoost) models. For the LR model, a stepwise method (both directions) was used for feature selection. For XGBoost, improvements in accuracy were used for the feature selection. After model building, 10‐fold cross‐validation^23^ was performed. The performance of all risk models was evaluated using the AUC statistics of the receiver operating characteristic (ROC). The cutoff value for the ROC curve was calculated using the Youden method, and the corresponding sensitivity and specificity were also calculated. The 95% confidence interval (CI) of the AUC was calculated by bootstrapping 2000 times. Two ROC curves were compared using bootstrap 2000 replicates, and the *p*‐value was calculated. Further stratifications were applied by histology, sex, smoking status (non‐smoker vs smoker), and family history.

All tests were two‐sided, and statistical significance was set at *p* < 0.05. All the above analyses were performed using R v3.6.2.

## 3 RESULTS

The epidemiologic characteristics of 974 patients with lung cancer and 1005 controls are shown in Table 1 and Table S1. Male patients (71.1%) enrolled more than females. ADC accounted for 48.8% and SCC for 36.5% of all lung cancer patients. Smoking intensity was significantly associated with lung cancer risk; however, smoking duration was not associated with lung cancer risk.

**TABLE 1:** Characteristics of lung cancer patients and controls in Chinese population

	All	Control	Case	
	1979	1005 (50.8)	974 (49.2)	
Sex				
Female	571 (28.9)	307 (53.8)	264 (46.2)	0.101^a^
Male	1408 (71.1)	698 (49.6)	710 (50.4)	
Age (mean ± SD)	62.21 ± 10.76	62.19 ± 10.74	62.23 ± 10.80	0.944^b^
Age (median)	62	63	62	
Age				
<60	753 (38.1)	382 (50.7)	371 (49.3)	0.993^a^
≥60	1225 (61.9)	623 (50.9)	602 (49.1)	
Smoking status				
Non‐smoker	782 (40.0)	503 (64.3)	279 (35.7)	<0.001^a^
Smoker	1174 (60.0)	502 (42.8)	672 (57.2)	
Smoking intensity (mean ± SD)	19.99 ± 11.25	15.28 ± 8.16	23.83 ± 11.96	<0.001^b^
Smoking intensity (median)	20	20	20	
Smoking duration (year) (mean ± SD)	35.35 ± 12.25	34.95 ± 12.18	35.67 ± 12.32	0.331^b^
Smoking duration (year) (median)	36	35	37	
Family history				
With family history	655 (33.1)	318 (48.5)	337 (51.5)	0.177^a^
Without family history	1324 (66.9)	687 (51.9)	637 (48.1)	

Abbreviation: SD, standard deviation.

^a^ Chi‐squared test *p*‐value.

^b^ *t*‐test *p*‐value.

All 61 SNPs were successfully genotyped. The association between 61 SNPs and lung cancer risk was evaluated in the additive model. After adjustment for sex and age, two SNPs were significantly associated with lung cancer risk (*p* < 0.05, Tables [Link], [Link]). TYMS rs3819102 conferred an increased risk of lung cancer in the additive model. BAG6 rs1077393 was found to decrease the risk of developing lung cancer. Further stratification showed that TYMS rs3819102 was significantly associated with the risk of SCC. Compared with the additive model, no new significant lung cancer‐associated SNPs were found in the dominant and recessive models. Some results have been previously published.^22^, ^24^

For lung cancer risk prediction, the model predicted only with epidemiology attained an AUC of 0.703 for LR and 0.744 for XGBoost (Table 2). Further addition of SNPs increased the AUC to 0.759 in the XGBoost model. Significant improvements were achieved using XGBoost and the addition of SNPs (*p* < 0.001).

**TABLE 2:** Combined lung cancer risk prediction AUC in overall study subjects

Model	Method	AUC^a^	AUC 95% CI	Sensitivity	Specificity	*p*
Epidemiology‐only model^b^	LR	0.703	0.681–0.726	0.562	0.732	Ref
	XGBoost	0.744	0.721–0.766	0.552	0.813	0.0112
Full model^c^	LR	0.742	0.718–0.765	0.663	0.699	0.0253
	XGBoost	0.759	0.737–0.782	0.638	0.746	<0.001

Abbreviations: AUC, area under the curve statistics of receiver operating characteristic; CI, confidence interval; LR, logistic regression; XGBoost, extreme gradient boosting.

^a^ AUC calculated by 10‐fold cross‐validation.

^b^ Epidemiology‐only model indicated model prediction without SNPs.

^c^ Full model indicated model prediction with SNPs.

The improvements in the accuracy of the model variables are listed in Table 3. Smoking intensity was the most important factor in the model, followed by smoking duration, age, and sex. Among the 17 SNPs included in the lung cancer model, BAG6 rs1077393, TERT rs2735845, and CAMKK1 rs7214723 were more important than family history. BAG6 rs1077393 and TERT rs2735845 were significantly associated with lung cancer risk in our study, while CAMKK1 rs7214723 showed no association with lung cancer risk.

**TABLE 3:** Improvement in accuracy for epidemiology factor and SNPs in the XGBoost‐base lung cancer risk model

Variable	Gain
Smoking intensity	0.340
Smoking duration	0.153
Age	0.124
Sex	0.037
BAG6 rs1077393	0.030
TERT rs2735845	0.028
CAMKK1 rs7214723	0.028
Family history	0.028
BAG6 rs1077394	0.027
TYMS rs3819102	0.024
MTHFR rs17037396	0.024
CHRNB3 rs16891561	0.023
IL1RAP rs4687163	0.017
XRCC6 rs2267437	0.016
ARHGEF11 rs868188	0.016
TGFBR2 rs3773663	0.016
ERCC2 rs1799793	0.014
GSTP1 rs1695	0.013
CHRNA6 rs16891604	0.013
BAG6 rs3130048	0.010
CHRNB3 rs4954	0.009
NQO1 rs1800566	0.009

We performed model building for ADC and SCC risks separately (Table 4). XGBoost was applied in the model for the ADC and significantly elevated model performance. Compared with the LR model predicted only with epidemiology, the XGBoost model predicted with epidemiology, and SNPs achieved a significant elevation in the AUC, of approximately 0.06 (*p* = 0.009). Further analysis of model accuracy improvement showed that age was more important than smoking duration in ADC (Table 5). Moreover, family history was not considered important in the ADC model. The three top SNPs in the ADC model were CHRNB3 rs4236926, ARHGEF11 rs868188, and NQO1 rs1800566. All three SNPs were not significantly associated with ADC risk in the logistic regression analysis.

**TABLE 4:** ADC and SCC risk prediction model

Model	Method	AUC^a^	AUC 95% CI	Sensitivity	Specificity	*p*
ADC						
Epidemiology‐only model^b^	LR	0.639	0.606–0.669	0.632	0.596	Ref
	XGBoost	0.683	0.652–0.714	0.638	0.666	<0.001
Full model^c^	LR	0.666	0.633–0.698	0.551	0.695	0.255
	XGBoost	0.699	0.666–0.733	0.739	0.567	0.009
SCC						
Epidemiology‐only model^b^	LR	0.818	0.786–0.847	0.786	0.737	Ref
	XGBoost	0.814	0.785–0.842	0.754	0.788	0.848
Full model^c^	LR	0.833	0.805–0.859	0.745	0.805	0.491
	XGBoost	0.816	0.782–0.847	0.762	0.745	0.893

Abbreviations: ADC, lung adenocarcinoma; AUC, area under the curve statistics of receiver operating characteristic; CI, confidence interval; LR, logistic regression; SCC, lung squamous cell carcinoma; XGBoost, extreme gradient boosting.

^a^ AUC calculated by 10‐fold cross‐validation.

^b^ Epidemiology‐only model indicated model prediction without SNPs.

^c^ Full model indicated model prediction with SNPs.

**TABLE 5:** Improvement in accuracy for epidemiology factor and SNPs in the XGBoost‐base lung adenocarcinoma risk model

Variable	Gain
Smoking intensity	0.236
Age	0.169
Smoking duration	0.107
Sex	0.065
CHRNB3 rs4236926	0.053
ARHGEF11 rs868188	0.040
NQO1 rs1800566	0.039
BAG6 rs1077394	0.038
TGFBR2 rs3773658	0.038
TGFBR2 rs9790292	0.036
TYMS rs3819102	0.032
MMP12 rs586701	0.024
MTHFR rs17037396	0.015
CHRNB3 rs16891561	0.014
TERT rs10069690	0.014
ERCC2 rs13181	0.013
CRP rs2808630	0.013
IL1B rs3136558	0.013
TGFBR2 rs3087465	0.012
TERT rs4246742	0.010
ERCC2 rs1799793	0.008
XPA rs1800975	0.008
BAG6 rs3130048	0.004

The AUC of all SCC models was above 0.8 (Table 4). The performance of the LR model predicted only with epidemiology was AUC = 0.818. Adding SNPs or applying XGBoost resulted in little improvement in AUC, with the best AUC being 0.833 in the LR model predicted with epidemiology and SNPs. The most important factors for the SCC model were smoking intensity, followed by smoking duration and age (Table 6). Family history had a relatively low importance in the SCC model. The most important SNPs in the SCC model were BAG6 rs3130048, NQO1 rs1800566, and TERT rs2735845.

**TABLE 6:** Improvement in accuracy for epidemiology factor and SNPs in the XGBoost‐base lung squamous cell carcinoma risk model

Variable	Gain
Smoking intensity	0.362
Smoking duration	0.134
Age	0.108
BAG6 rs3130048	0.038
NQO1 rs1800566	0.028
TERT rs2735845	0.027
TYMS rs3819102	0.027
CHEK2 rs2236141	0.023
BAG6 rs1077393	0.021
CHRNB3 rs4954	0.021
TGFBR2 rs3773663	0.019
TGFBR2 rs3773658	0.019
IL1RAP rs4687163	0.018
CHRNA6 rs16891604	0.018
Family history	0.017
CHRNB3 rs16891561	0.017
TERT rs2075786	0.017
BAG6 rs1077394	0.016
CRP rs2808630	0.016
MMP2 rs243865	0.015
TERT rs2853676	0.015
MTHFR rs17037396	0.013
TERT rs4246742	0.011

In the male population (AUC = 0.791), people aged 60 and older (AUC = 0.761), smoking (AUC = 0.785), people without family history (AUC = 0.734), and people with family history (AUC = 0.790), lung cancer risk prediction showed good performance. Interestingly, some of these sub‐populations (male, people aged 60 and older, smoker, and people with family history) showed that the SNP‐based model predicted with epidemiology and SNPs built by XGBoost is the best approach for modeling, while the others (female, people under age 60, non‐smoker, people without family history) showed that LR is the best approach for modeling. All the models in stratification showed a trend of improved model performance in the LR model before and after adding SNPs, in which some models were significant (female, non‐smoker) (Table 7).

**TABLE 7:** Lung cancer risk prediction model in stratification

Model	Method	AUC^a^	AUC 95% CI	Sensitivity	Specificity	*p*
Male						
Epidemiology‐only model^b^	LR	0.745	0.720–0.770	0.754	0.637	Ref
	XGBoost	0.782	0.757–0.806	0.603	0.829	<0.001
Full model^c^	LR	0.775	0.749–0.801	0.688	0.718	0.107
	XGBoost	0.791	0.766–0.815	0.664	0.787	0.016
Female						
Epidemiology‐only model^b^	LR	0.537	0.490–0.585	0.548	0.518	ref
	XGBoost	0.547	0.497–0.599	0.277	0.827	0.785
Full model^c^	LR	0.644	0.596–0.690	0.638	0.627	0.002
	XGBoost	0.614	0.561–0.664	0.724	0.452	0.033
Age <60						
Epidemiology‐only model^b^	LR	0.665	0.623–0.704	0.607	0.690	ref
	XGBoost	0.652	0.611–0.691	0.619	0.635	0.483
Full model^c^	LR	0.720	0.680–0.763	0.582	0.749	0.052
	XGBoost	0.718	0.675–0.759	0.564	0.785	0.080
Age ≥60						
Epidemiology‐only model^b^	LR	0.734	0.707–0.762	0.612	0.727	Ref
	XGBoost	0.761	0.733–0.786	0.634	0.731	0.002
Full model^c^	LR	0.750	0.720–0.777	0.724	0.637	0.452
	XGBoost	0.761	0.733–0.789	0.724	0.650	0.184
Non‐smoker						
Epidemiology‐only model^b^	LR	0.626	0.587–0.665	0.705	0.557	ref
	XGBoost	0.614	0.575–0.653	0.777	0.471	0.389
Full model^c^	LR	0.692	0.651–0.734	0.676	0.616	0.022
	XGBoost	0.667	0.623–0.708	0.583	0.691	0.160
Smoker						
Epidemiology‐only model^b^	LR	0.736	0.707–0.766	0.524	0.836	Ref
	XGBoost	0.762	0.734–0.789	0.631	0.766	0.012
Full model^c^	LR	0.756	0.726–0.785	0.638	0.737	0.331
	XGBoost	0.785	0.757–0.813	0.623	0.806	0.014
Without family history						
Epidemiology‐only model^b^	LR	0.701	0.672–0.729	0.432	0.855	ref
	XGBoost	0.702	0.671–0.731	0.402	0.911	0.954
Full model^c^	LR	0.740	0.712–0.769	0.758	0.607	0.067
	XGBoost	0.734	0.706–0.764	0.646	0.706	0.116
With family history						
Epidemiology‐only model^b^	LR	0.761	0.724–0.797	0.877	0.532	ref
	XGBoost	0.755	0.716–0.793	0.801	0.614	0.828
Full model^c^	LR	0.777	0.740–0.813	0.672	0.754	0.557
	XGBoost	0.790	0.751–0.826	0.794	0.684	0.284

Abbreviations: AUC, area under the curve statistics of receiver operating characteristic; CI, confidence interval; LR, logistic regression; XGBoost, extreme gradient boosting.

^a^ AUC calculated by 10‐fold cross‐validation.

^b^ Epidemiology‐only model indicated model prediction without SNPs.

^c^ Full model indicated model prediction with SNPs.

In different stratifications, the clinical factors are different. For the lung cancer risk model in the male population, people under age 60, people aged 60 and older, smokers, and people without family history, the top three important factors were smoking intensity, smoking duration, and age. For the lung cancer risk model in people with family history and age was a more important factor than smoking duration. For the lung cancer risk model in the female population, age was the most important factor, while smoking duration became less important.

In all stratifications of the models, TYMS rs3819102, the most significant SNP associated with lung cancer (*p* = 0.007), was used. This SNP was found to have an important role (among the top five important SNPs) in risk models for males, people aged 60 and older, smokers, and people without family history. In the lung cancer risk model, BAG6 rs1077393, the most important SNP, remained in the top five important SNPs in risk models for people under the age of 60 and people with family history.

## 4 DISCUSSION

This study demonstrates the use of logistic regression and XGBoost algorithms to investigate the contribution of SNPs to the prediction of lung cancer susceptibility. Using genotype data from 974 lung cancer patients and 1004 healthy people in the Chinese population, we found that adding SNPs or applying the XGBoost algorithm to the epidemiologic‐based logistic regression risk prediction model significantly improved the model performance, especially for the lung cancer risk prediction model and ADC risk prediction model.

Lung cancer is a heterogeneous disease that includes two main types: ADC (40%) and SCC (25–30%). Different disease patterns and treatment strategies exist.^25^ Exome sequences in tumor‐normal pairs reveal that ADC and SCC are less similar than other cancer types for significantly mutated genes.^26^ GWAS in Caucasians also revealed that SNPs associated with both ADC and SCC only account for a small part of ADC‐ or SCC‐related SNPs.^27^ However, heterogeneity between ADC and SCC has not been considered in model‐building studies in the Chinese population.^9^, ^10^, ^11^ Our model has a better result for lung cancer models when compared with previous model‐building studies in the Chinese population.

Smoking intensity is the most important factor that improves the performance of the XGBoost model in the lung cancer risk model. Cigarette smoke (CS) contains hundreds of carcinogens and various factors that induce cancer. CS‐generated oxidants lead to DNA adducts and further induce DNA double‐strand breaks (DSBs) in normal human bronchial epithelial cells or A549 pulmonary carcinoma cells, especially in S‐phase cells.^28^ Decreased DNA repair protein XPC expression impairs oxidative DNA damage repair and promotes lung ADC development in response to CS‐carcinogen exposure.^29^ Another in vitro study showed that cigarette smoke components notably inhibited glycogen synthase kinase 3 (GSK3) and induced the expression of involucrin, a marker of squamous differentiation, implying a possible mechanism for CS‐induced squamous differentiation.^30^

We found good predictive performance in the SCC model, with logistic regression, whereas SNPs played little role in the model. Smoking is a major risk factor for lung cancer,^31^, ^32^ and smokers mostly develop squamous cell lung cancer with a high somatic mutation burden.^33^ Our SCC model has a good effect, suggesting that smoking plays a key role in the development of lung SCC. The role of SNPs in the model is relatively small, possibly because the environment‐induced somatic mutations in lung SCC exceed the role of germline SNPs.

In our study population, TYMS rs3819102 increased lung cancer risk in our study population and also showed importance in our lung cancer risk prediction models, especially among the population of males, people aged 60 and older, smokers, and people without a family history of cancer. TYMS encodes thymidylate synthase, an enzyme involved in the biosynthesis of thymidylate, and is a key regulator of DNA synthesis.^34^ Thymidylate synthase uses CH2H4 folate as a methylene group donor; thus, the function of this enzyme is regulated by the folate pathway. The TCGA database shows that TYMS expression is elevated in lung ADC and SCC.^35^ Data from three microarray datasets and protein–protein interaction network construction show that TYMS expression changes are the key signature in carcinogenesis of NSCLC.^36^ rs3819102, located in the 3′‐flanking region of TYMS, was reported to increase the risk of endometrial cancer in the Chinese population.^21^

In both XGBoost‐based lung cancer risk and SCC risk prediction models, the most important SNP was in BAG6. BAG6 is involved in cellular processes, such as apoptosis, gene regulation, and protein degradation.^37^ Soluble BAG6 can be released from heat‐shocked tumor cells and inhibit NK cell cytotoxicity, indicating that BAG6 may be a mediator of tumor immune escape. Polymorphism in BAG6 (rs3117582) was reported to have strong evidence of association with lung cancer risk among Caucasians in a meta‐analysis.^38^ However, in the ADC risk prediction model, CHRNB3 was found to be more important than BAG6. CHRNB3 is a kind of nicotinic acetylcholine receptor, and a study among Caucasian smokers involving 661 lung ADC cases and 1347 controls found that two CHRNA3 SNPs (rs1051730 and rs12914385) associated with ADC risk had significant indirect effects on lung ADC risk through nicotine dependence.^39^

There are several points in our research that require improvement. First, before applying this model, it was necessary to verify the effect of the model in a larger cohort. Next, the predictive performance of the ADC model was poor, the main reason for which may be that at the time of SNP selection, most studies focused on the effect of SNPs on lung cancer and smoking; only a small part of the study reported ADC‐associated SNPs, so most SNPs included in this study were associated with smoking. Future studies could include more ADC‐associated SNPs in the model. Moreover, environmental factors such as particulate matter 2.5 level^40^ were not included in the model. Thus, more SNPs and environmental factors need to be considered in the model and this result should be validated with independent studies or meta‐analysis. In summary, our XGBoost modeling method significantly improved the predictive performance of the lung cancer model in the Chinese population. This model may be useful in assisting the early screening of lung cancer, which develops clinical application of machine learning in genetic epidemiological data for screening and surveillance. High‐risk population such as people with family history or somkers whose risk is above the cutoff value should receive lung cancer screening examinations with LDCT^41^, ^42^, ^43^ and have access to high‐quality lung cancer screening early. Moreover, the SNPs included in our model may provide new targets for lung cancer.

## ETHICS STATEMENT

The studies involving human participants were reviewed and approved by the ethics committee of the School of Life Sciences, Fudan University. The patients/participants provided their written informed consent to participate in this study.

## CONFLICT OF INTEREST

No conflict of interest exits in the submission of this manuscript. All the authors have contributed to, read and approved the final manuscript for submission. This manuscript has not been submitted elsewhere.

## Supporting information


Table S1

Table S2

Table S3

Table S4

Table S5

Table S6

Table S7

## Data Availability

Availability of data and materials #10;The datasets used during the current study are available from the corresponding author on reasonable request.
