# Supplementary material for: Prediction of lung cancer risk in Chinese population with genetic‐environment factor using extreme gradient boosting
Source: Cancer Med. 2022 May 2;11(23):4469–78. doi: 10.1002/cam4.4800 (PMC9741969; doi:10.1002/cam4.4800)
Supplement: Supplementary file 1 — Table S1 [file CAM4-11-4469-s005.docx]

**Supplementary Table1. Characteristics for all lung cancer patients**

|  | All case | Shanghai | Taizhou |
| --- | --- | --- | --- |
|  | 974 | 622 (63.9) | 352 (36.1) |
| Sex |  |  |  |
| Female | 264 (27.1) | 180 (68.2) | 84 (31.8) |
| Male | 710 (72.9) | 442 (62.3) | 268 (37.7) |
| Age (mean±SD) | 62.23 ± 10.80 | 61.23 ± 11.14 | 63.99 ± 9.94 |
| Age (median) | 62 | 61 | 65 |
| Age |  |  |  |
| Age<60 | 371 (38.1) | 263 (70.9) | 108 (29.1) |
| Age>=60 | 602 (61.9) | 358 (59.5) | 244 (40.5) |
| Smoking status |  |  |  |
| Non smoker | 279 (29.3) | 194 (69.5) | 85 (30.5) |
| Smoker | 672 (70.7) | 406 (60.4) | 266 (39.6) |
| Smoking intensity (mean±SD) | 23.83 ± 11.96 | 25.81 ± 13.51 | 21.27 ± 8.99 |
| Smoking intensity (median) | 20 | 20 | 20 |
| Smoking time (Year) (mean±SD) | 35.67 ± 12.32 | 32.64 ± 12.43 | 39.59 ± 11.01 |
| Smoking time (Year) (median) | 37 | 30 | 40 |
| Family history |  |  |  |
| With family history | 337 (34.6) | 194 (57.6) | 143 (42.4) |
| Without family history | 637 (65.4) | 428 (67.2) | 209 (32.8) |
| Subtype |  |  |  |
| SCLC | 83 (8.5) | 45 (54.2) | 38 (45.8) |
| NSCLC | 891 (91.5) | 577 (64.8) | 314 (35.2) |
| ADC | 435 (48.8) | 319 (73.3) | 116 (26.7) |
| SCC | 325 (36.5) | 179 (55.1) | 146 (44.9) |
| Other | 131 (14.7) | 79 (60.3) | 52 (39.7) |

SD: standard deviation

SCLC: small cell lung cancer

NSCLC: non-small cell lung cancer

ADC: lung adenocarcinoma

SCC: lung squamous cell carcinoma
