# Supplementary material for: Prediction of lung cancer risk in Chinese population with genetic‐environment factor using extreme gradient boosting
Source: Cancer Med. 2022 May 2;11(23):4469–78. doi: 10.1002/cam4.4800 (PMC9741969; doi:10.1002/cam4.4800)
Supplement: Supplementary file 2 — Table S2 [file CAM4-11-4469-s003.docx]

**Supplementary Table 2 Character of SNPs genotyped in this study**

| **Gene Group** | **Gene** | **SNP** | **Ref Allele** | **Mut Allele** | **Wild type** | **Heterozygous mutant** | **Homozygous Mutant** | **MAF** | **HWE** | **Call rate (%)** |
| --- | --- | --- | --- | --- | --- | --- | --- | --- | --- | --- |
| **metabolism** | **CRP** | **rs2808630** | T | C | 1395 | 528 | 56 | 0.162 | 0.100 | 100.0 |
|  | **EPHX1** | **rs1051741** | C | T | 1599 | 345 | 17 | 0.096 | 0.403 | 99.1 |
|  | **EPHX1** | **rs2292568** | C | T | 1477 | 460 | 40 | 0.136 | 0.407 | 99.9 |
|  | **GSTP1** | **rs1695** | A | G | 1317 | 590 | 70 | 0.184 | 0.454 | 99.9 |
|  | **IL1B** | **rs12621220** | C | T | 713 | 938 | 324 | 0.401 | 0.767 | 99.8 |
|  | **IL1B** | **rs1143623** | C | G | 711 | 932 | 330 | 0.402 | 0.768 | 99.7 |
|  | **IL1B** | **rs16944** | G | A | 562 | 960 | 455 | 0.472 | 0.429 | 99.9 |
|  | **IL1B** | **rs3136558** | A | G | 745 | 895 | 287 | 0.371 | 0.297 | 97.4 |
|  | **IL1B** | **rs1143627** | A | G | 535 | 932 | 458 | 0.467 | 0.463 | 97.3 |
|  | **IL1RAP** | **rs4687163** | A | G | 1296 | 574 | 66 | 0.178 | 0.234 | 97.8 |
|  | **MTHFR** | **rs17037396** | C | T | 1611 | 345 | 23 | 0.099 | 0.336 | 100.0 |
|  | **MTHFR** | **rs1801133** | G | A | 592 | 935 | 400 | 0.438 | 0.587 | 97.4 |
|  | **NQO1** | **rs1800566** | G | A | 566 | 969 | 439 | 0.467 | 0.681 | 99.7 |
| **extracellular component** | **MMP12** | **rs586701** | T | G | 1446 | 490 | 43 | 0.146 | 0.638 | 100.0 |
|  | **MMP2** | **rs2285053** | C | T | 1183 | 680 | 112 | 0.228 | 0.269 | 99.8 |
|  | **MMP2** | **rs243865** | C | T | 1513 | 390 | 23 | 0.110 | 0.570 | 97.3 |
|  | **MMP9** | **rs2250889** | C | G | 1063 | 784 | 125 | 0.261 | 0.904 | 99.6 |
| **DNA repair** | **ERCC2** | **rs1799793** | C | T | 1730 | 240 | 8 | 0.065 | 0.678 | 99.9 |
|  | **ERCC2** | **rs13181** | T | G | 1651 | 278 | 9 | 0.075 | 0.267 | 97.9 |
|  | **XPA** | **rs1800975** | C | T | 495 | 983 | 488 | 0.495 | 0.394 | 99.3 |
|  | **XRCC6** | **rs2267437** | C | G | 1250 | 573 | 102 | 0.196 | 0.024 | 97.3 |
| **cell singaling** | **ARHGEF11** | **rs868188** | T | C | 508 | 1020 | 420 | 0.470 | 0.190 | 98.4 |
|  | **CAMKK1** | **rs7214723** | T | C | 829 | 890 | 245 | 0.349 | 0.035 | 99.2 |
|  | **CHRNA6** | **rs16891604** | C | A | 1326 | 566 | 81 | 0.184 | 0.104 | 99.7 |
|  | **CHRNA6** | **rs9298628** | C | T | 1177 | 700 | 102 | 0.228 | 0.167 | 100.0 |
|  | **CHRNB3** | **rs16891569** | C | T | 1651 | 310 | 8 | 0.082 | 0.284 | 99.5 |
|  | **CHRNB3** | **rs4954** | A | G | 1461 | 464 | 41 | 0.138 | 0.399 | 99.3 |
|  | **CHRNB3** | **rs16891561** | C | T | 1202 | 679 | 95 | 0.220 | 0.099 | 99.8 |
|  | **CHRNB3** | **rs4236926** | G | T | 1200 | 680 | 98 | 0.221 | 0.099 | 99.9 |
|  | **EGFR** | **rs763317** | G | A | 1258 | 623 | 85 | 0.200 | 0.661 | 99.3 |
|  | **TGFBR2** | **rs3087465** | G | A | 1342 | 563 | 71 | 0.178 | 0.958 | 99.8 |
|  | **TGFBR2** | **rs2228048** | C | T | 1076 | 739 | 162 | 0.269 | 0.022 | 99.9 |
|  | **TGFBR2** | **rs3773658** | A | G | 986 | 817 | 176 | 0.295 | 0.376 | 100.0 |
|  | **TGFBR2** | **rs9790292** | C | T | 788 | 874 | 301 | 0.373 | 0.060 | 99.2 |
|  | **TGFBR2** | **rs3773663** | G | A | 540 | 967 | 472 | 0.483 | 0.094 | 100.0 |
| **DNA synthesis** | **TERT** | **rs6554759** | A | G | 1797 | 172 | 10 | 0.049 | 0.221 | 100.0 |
|  | **TERT** | **rs2736122** | G | A | 1745 | 230 | 4 | 0.060 | 0.689 | 100.0 |
|  | **TERT** | **rs4635969** | G | A | 1607 | 355 | 17 | 0.098 | 0.558 | 100.0 |
|  | **TERT** | **rs4975605** | C | A | 1602 | 351 | 22 | 0.100 | 0.933 | 99.8 |
|  | **TERT** | **rs2075786** | A | G | 1374 | 510 | 46 | 0.152 | 0.390 | 97.5 |
|  | **TERT** | **rs10069690** | C | T | 1293 | 549 | 64 | 0.171 | 0.869 | 96.3 |
|  | **TERT** | **rs2853676** | C | T | 1311 | 587 | 63 | 0.180 | 0.637 | 99.1 |
|  | **TERT** | **rs2735845** | C | G | 829 | 887 | 258 | 0.354 | 0.379 | 99.7 |
|  | **TERT** | **rs4246742** | T | A | 822 | 902 | 255 | 0.357 | 0.917 | 100.0 |
|  | **TERT** | **rs2853668** | G | T | 879 | 853 | 188 | 0.311 | 0.793 | 97.0 |
|  | **TYMS** | **rs3819102** | A | G | 1137 | 700 | 128 | 0.242 | 0.287 | 99.3 |
| **Apoptosis** | **BAG6** | **rs3130628** | T | C | 1495 | 400 | 23 | 0.113 | 0.190 | 96.9 |
|  | **BAG6** | **rs3130047** | G | A | 1525 | 431 | 22 | 0.120 | 0.152 | 99.9 |
|  | **BAG6** | **rs805298** | C | A | 1509 | 437 | 32 | 0.127 | 0.734 | 99.9 |
|  | **BAG6** | **rs2077102** | G | T | 1442 | 493 | 43 | 0.146 | 0.407 | 99.9 |
|  | **BAG6** | **rs2242656** | A | G | 1379 | 543 | 57 | 0.166 | 0.791 | 100.0 |
|  | **BAG6** | **rs9380266** | T | G | 1185 | 688 | 101 | 0.225 | 0.821 | 99.7 |
|  | **BAG6** | **rs1077394** | A | G | 945 | 823 | 206 | 0.312 | 0.029 | 99.7 |
|  | **BAG6** | **rs3130048** | A | G | 844 | 883 | 247 | 0.348 | 0.651 | 99.7 |
|  | **BAG6** | **rs1077393** | G | A | 660 | 878 | 360 | 0.404 | 0.459 | 95.9 |
|  | **BAG6** | **rs1052486** | C | T | 646 | 948 | 376 | 0.430 | 0.388 | 99.5 |
|  | **BAG6** | **rs2844463** | G | A | 1027 | 756 | 144 | 0.264 | 0.725 | 97.4 |
|  | **CHEK2** | **rs2236141** | C | T | 1394 | 485 | 48 | 0.147 | 0.949 | 97.4 |
|  | **CLPTM1L** | **rs31489** | C | A | 1460 | 472 | 44 | 0.141 | 0.278 | 99.8 |
|  | **CLPTM1L** | **rs402710** | C | T | 1007 | 777 | 188 | 0.291 | 0.139 | 99.6 |
|  | **RBMS3** | **rs1530057** | G | T | 1616 | 337 | 21 | 0.096 | 0.309 | 99.7 |

SNP: single nucleotide polymorphism

MAF: minor allele frequency

HWE: Hardy-Weinberg equilibrium p-value
