# Supplementary material for: Prediction of lung cancer risk in Chinese population with genetic‐environment factor using extreme gradient boosting
Source: Cancer Med. 2022 May 2;11(23):4469–78. doi: 10.1002/cam4.4800 (PMC9741969; doi:10.1002/cam4.4800)
Supplement: Supplementary file 3 — Table S3 [file CAM4-11-4469-s001.docx]

**Supplementary Table 3 Association between lung cancer and SNPs in additive model**

|  |  | Lung cancer | |  | ADC | |  | SCC | |
| --- | --- | --- | --- | --- | --- | --- | --- | --- | --- |
| Gene | SNP | OR (95% CI) ^a^ | P ^a^ |  | OR (95% CI) ^a^ | P ^a^ |  | OR (95% CI) ^a^ | P ^a^ |
| ARHGEF11 | rs868188 | 1.053 (0.925-1.198) | 0.825 |  | 1.149 (0.976-1.353) | 0.553 |  | 0.950 (0.787-1.148) | 0.974 |
| BAG6 | rs3130628 | 0.985 (0.807-1.202) | 0.945 |  | 1.040 (0.802-1.342) | 0.854 |  | 0.939 (0.698-1.251) | 0.974 |
| BAG6 | rs3130047 | 1.031 (0.848-1.253) | 0.913 |  | 1.127 (0.878-1.440) | 0.729 |  | 0.887 (0.661-1.179) | 0.913 |
| BAG6 | rs805298 | 0.878 (0.727-1.060) | 0.511 |  | 1.011 (0.798-1.274) | 0.973 |  | 0.800 (0.598-1.057) | 0.504 |
| BAG6 | rs2077102 | 1.030 (0.863-1.228) | 0.913 |  | 0.950 (0.752-1.195) | 0.854 |  | 1.033 (0.801-1.324) | 0.974 |
| BAG6 | rs2242656 | 0.804 (0.679-0.950) | 0.222 |  | 0.905 (0.730-1.117) | 0.729 |  | 0.711 (0.547-0.914) | 0.093 |
| BAG6 | rs9380266 | 1.009 (0.869-1.172) | 0.954 |  | 1.000 (0.822-1.213) | 0.999 |  | 1.050 (0.848-1.295) | 0.974 |
| BAG6 | rs1077394 | 0.873 (0.764-0.998) | 0.272 |  | 0.903 (0.761-1.071) | 0.698 |  | 0.840 (0.690-1.020) | 0.378 |
| BAG6 | rs3130048 | 1.017 (0.893-1.158) | 0.913 |  | 1.041 (0.880-1.231) | 0.854 |  | 0.984 (0.815-1.186) | 0.974 |
| BAG6 | rs1077393 | 0.811 (0.714-0.920) | 0.036 |  | 0.871 (0.739-1.026) | 0.553 |  | 0.760 (0.628-0.917) | 0.093 |
| BAG6 | rs1052486 | 0.923 (0.814-1.045) | 0.523 |  | 0.982 (0.836-1.153) | 0.886 |  | 0.834 (0.694-1.002) | 0.325 |
| BAG6 | rs2844463 | 0.879 (0.762-1.013) | 0.331 |  | 0.950 (0.789-1.140) | 0.827 |  | 0.781 (0.629-0.963) | 0.196 |
| CAMKK1 | rs7214723 | 0.980 (0.860-1.117) | 0.913 |  | 0.925 (0.782-1.093) | 0.729 |  | 1.011 (0.839-1.216) | 0.974 |
| CHEK2 | rs2236141 | 1.111 (0.932-1.324) | 0.544 |  | 0.929 (0.732-1.173) | 0.806 |  | 1.397 (1.092-1.781) | 0.093 |
| CHRNA6 | rs16891604 | 0.823 (0.702-0.964) | 0.249 |  | 0.882 (0.720-1.077) | 0.698 |  | 0.712 (0.555-0.905) | 0.093 |
| CHRNA6 | rs9298628 | 0.995 (0.857-1.154) | 0.976 |  | 0.876 (0.720-1.063) | 0.620 |  | 1.091 (0.882-1.344) | 0.913 |
| CHRNB3 | rs16891569 | 0.844 (0.668-1.064) | 0.482 |  | 0.885 (0.652-1.190) | 0.729 |  | 0.794 (0.559-1.110) | 0.671 |
| CHRNB3 | rs4954 | 0.952 (0.795-1.139) | 0.878 |  | 0.818 (0.641-1.037) | 0.553 |  | 1.121 (0.868-1.439) | 0.913 |
| CHRNB3 | rs16891561 | 0.894 (0.769-1.040) | 0.482 |  | 0.867 (0.712-1.051) | 0.564 |  | 0.972 (0.781-1.203) | 0.974 |
| CHRNB3 | rs4236926 | 0.909 (0.782-1.056) | 0.523 |  | 0.853 (0.700-1.035) | 0.553 |  | 0.973 (0.784-1.202) | 0.974 |
| CLPTM1L | rs31489 | 0.952 (0.797-1.137) | 0.878 |  | 0.967 (0.767-1.211) | 0.854 |  | 0.962 (0.739-1.242) | 0.974 |
| CLPTM1L | rs402710 | 0.979 (0.856-1.120) | 0.913 |  | 0.945 (0.794-1.123) | 0.806 |  | 0.945 (0.773-1.153) | 0.974 |
| CRP | rs2808630 | 1.141 (0.964-1.351) | 0.477 |  | 1.091 (0.879-1.351) | 0.729 |  | 1.252 (0.988-1.580) | 0.333 |
| EGFR | rs763317 | 0.983 (0.841-1.147) | 0.913 |  | 0.976 (0.799-1.188) | 0.881 |  | 0.923 (0.732-1.158) | 0.942 |
| EPHX1 | rs1051741 | 1.082 (0.874-1.339) | 0.857 |  | 1.047 (0.790-1.378) | 0.854 |  | 0.983 (0.713-1.339) | 0.974 |
| EPHX1 | rs2292568 | 0.878 (0.732-1.051) | 0.482 |  | 0.926 (0.733-1.163) | 0.806 |  | 0.897 (0.688-1.158) | 0.913 |
| ERCC2 | rs1799793 | 1.141 (0.885-1.473) | 0.652 |  | 1.303 (0.950-1.775) | 0.553 |  | 0.850 (0.568-1.241) | 0.913 |
| ERCC2 | rs13181 | 1.268 (0.998-1.614) | 0.272 |  | 1.158 (0.842-1.578) | 0.729 |  | 1.124 (0.787-1.585) | 0.945 |
| GSTP1 | rs1695 | 0.851 (0.724-0.999) | 0.272 |  | 0.825 (0.668-1.014) | 0.553 |  | 0.883 (0.696-1.113) | 0.871 |
| IL1B | rs12621220 | 1.042 (0.917-1.182) | 0.874 |  | 1.031 (0.875-1.213) | 0.854 |  | 1.072 (0.892-1.286) | 0.942 |
| IL1B | rs1143623 | 1.015 (0.894-1.151) | 0.913 |  | 1.053 (0.894-1.238) | 0.806 |  | 1.033 (0.860-1.240) | 0.974 |
| IL1B | rs16944 | 1.022 (0.903-1.156) | 0.913 |  | 1.066 (0.909-1.250) | 0.729 |  | 0.993 (0.830-1.188) | 0.974 |
| IL1B | rs3136558 | 0.988 (0.868-1.125) | 0.936 |  | 0.962 (0.814-1.135) | 0.854 |  | 1.009 (0.838-1.212) | 0.974 |
| IL1B | rs1143627 | 1.025 (0.905-1.162) | 0.913 |  | 1.068 (0.908-1.257) | 0.729 |  | 0.986 (0.823-1.180) | 0.974 |
| IL1RAP | rs4687163 | 0.929 (0.789-1.093) | 0.760 |  | 0.845 (0.681-1.044) | 0.553 |  | 1.018 (0.805-1.281) | 0.974 |
| MMP12 | rs586701 | 1.195 (1.002-1.428) | 0.272 |  | 1.261 (1.006-1.576) | 0.553 |  | 1.144 (0.884-1.471) | 0.871 |
| MMP2 | rs2285053 | 0.966 (0.834-1.119) | 0.913 |  | 0.966 (0.798-1.165) | 0.854 |  | 1.058 (0.856-1.302) | 0.974 |
| MMP2 | rs243865 | 1.070 (0.875-1.308) | 0.864 |  | 1.227 (0.953-1.573) | 0.553 |  | 1.037 (0.769-1.385) | 0.974 |
| MMP9 | rs2250889 | 0.924 (0.800-1.068) | 0.623 |  | 0.837 (0.692-1.009) | 0.553 |  | 0.998 (0.808-1.229) | 0.985 |
| MTHFR | rs17037396 | 0.976 (0.793-1.200) | 0.913 |  | 1.006 (0.767-1.311) | 0.979 |  | 1.007 (0.739-1.355) | 0.982 |
| MTHFR | rs1801133 | 0.883 (0.778-1.002) | 0.272 |  | 0.910 (0.773-1.070) | 0.698 |  | 0.827 (0.688-0.992) | 0.316 |
| NQO1 | rs1800566 | 0.957 (0.845-1.084) | 0.857 |  | 1.006 (0.858-1.179) | 0.973 |  | 0.936 (0.779-1.124) | 0.942 |
| RBMS3 | rs1530057 | 1.060 (0.859-1.309) | 0.878 |  | 1.050 (0.799-1.370) | 0.854 |  | 0.864 (0.619-1.190) | 0.913 |
| TERT | rs6554759 | 1.042 (0.785-1.382) | 0.913 |  | 0.936 (0.639-1.344) | 0.854 |  | 1.113 (0.725-1.668) | 0.974 |
| TERT | rs2736122 | 0.910 (0.697-1.188) | 0.857 |  | 0.925 (0.651-1.295) | 0.854 |  | 0.939 (0.633-1.366) | 0.974 |
| TERT | rs4635969 | 0.852 (0.688-1.052) | 0.482 |  | 0.895 (0.678-1.171) | 0.729 |  | 0.834 (0.604-1.138) | 0.844 |
| TERT | rs4975605 | 0.917 (0.745-1.128) | 0.809 |  | 0.809 (0.608-1.064) | 0.553 |  | 1.042 (0.768-1.398) | 0.974 |
| TERT | rs2075786 | 0.839 (0.704-1.000) | 0.272 |  | 0.964 (0.774-1.196) | 0.854 |  | 0.769 (0.589-0.996) | 0.325 |
| TERT | rs10069690 | 0.998 (0.846-1.178) | 0.996 |  | 1.125 (0.914-1.381) | 0.698 |  | 0.866 (0.670-1.110) | 0.844 |
| TERT | rs2853676 | 0.956 (0.812-1.125) | 0.878 |  | 0.942 (0.762-1.161) | 0.827 |  | 0.990 (0.783-1.245) | 0.974 |
| TERT | rs2735845 | 1.165 (1.023-1.326) | 0.257 |  | 1.093 (0.925-1.291) | 0.717 |  | 1.278 (1.062-1.537) | 0.093 |
| TERT | rs4246742 | 1.127 (0.990-1.284) | 0.331 |  | 1.209 (1.025-1.427) | 0.496 |  | 1.031 (0.851-1.247) | 0.974 |
| TERT | rs2853668 | 1.149 (1.002-1.319) | 0.272 |  | 1.129 (0.946-1.347) | 0.620 |  | 1.195 (0.980-1.455) | 0.378 |
| TGFBR2 | rs3087465 | 0.971 (0.826-1.140) | 0.913 |  | 0.931 (0.752-1.148) | 0.806 |  | 0.981 (0.772-1.240) | 0.974 |
| TGFBR2 | rs2228048 | 1.000 (0.871-1.148) | 1.000 |  | 1.143 (0.961-1.357) | 0.553 |  | 0.839 (0.682-1.028) | 0.406 |
| TGFBR2 | rs3773658 | 1.089 (0.950-1.248) | 0.523 |  | 1.074 (0.900-1.280) | 0.729 |  | 1.149 (0.944-1.395) | 0.621 |
| TGFBR2 | rs9790292 | 1.104 (0.973-1.253) | 0.477 |  | 1.214 (1.034-1.425) | 0.496 |  | 0.959 (0.796-1.154) | 0.974 |
| TGFBR2 | rs3773663 | 1.081 (0.955-1.223) | 0.523 |  | 1.091 (0.931-1.277) | 0.717 |  | 1.066 (0.891-1.276) | 0.942 |
| TYMS | rs3819102 | 1.332 (1.153-1.541) | 0.007 |  | 1.284 (1.065-1.546) | 0.496 |  | 1.501 (1.224-1.839) | 0.005 |
| XPA | rs1800975 | 1.033 (0.911-1.171) | 0.892 |  | 1.069 (0.912-1.254) | 0.729 |  | 0.992 (0.827-1.189) | 0.974 |
| XRCC6 | rs2267437 | 0.910 (0.781-1.059) | 0.523 |  | 0.892 (0.729-1.087) | 0.698 |  | 0.910 (0.726-1.133) | 0.913 |

^a^ p-values were calculated in in multivariate logistic regression (adjust for sex, age) after false discovery rate (FDR) adjustment for multiple testing

SNP: single nucleotide polymorphism

OR: odds ratio

CI: confidence interval

ADC: lung adenocarcinoma

SCC: lung squamous cell carcinoma
