# Supplementary material for: Prediction of lung cancer risk in Chinese population with genetic‐environment factor using extreme gradient boosting
Source: Cancer Med. 2022 May 2;11(23):4469–78. doi: 10.1002/cam4.4800 (PMC9741969; doi:10.1002/cam4.4800)
Supplement: Supplementary file 4 — Table S4 [file CAM4-11-4469-s002.docx]

**Supplementary Table 4 Association between lung cancer and SNPs in dominant model**

|  |  | Lung cancer | |  | ADC | |  | SCC | |
| --- | --- | --- | --- | --- | --- | --- | --- | --- | --- |
| Gene | SNP | OR (95% CI) ^a^ | P ^a^ |  | OR (95% CI) ^a^ | P ^a^ |  | OR (95% CI) ^a^ | P ^a^ |
| ARHGEF11 | rs868188 | 1.087 (0.888-1.331) | 0.784 |  | 1.093 (0.844-1.422) | 0.841 |  | 1.040 (0.779-1.398) | 0.927 |
| BAG6 | rs3130628 | 0.953 (0.767-1.182) | 0.901 |  | 1.037 (0.787-1.360) | 0.985 |  | 0.887 (0.640-1.217) | 0.819 |
| BAG6 | rs3130047 | 1.012 (0.820-1.249) | 0.959 |  | 1.113 (0.852-1.448) | 0.841 |  | 0.848 (0.616-1.156) | 0.803 |
| BAG6 | rs805298 | 0.880 (0.714-1.083) | 0.599 |  | 1.031 (0.793-1.335) | 0.985 |  | 0.787 (0.573-1.070) | 0.541 |
| BAG6 | rs2077102 | 1.063 (0.872-1.297) | 0.901 |  | 0.979 (0.755-1.265) | 0.985 |  | 1.097 (0.821-1.457) | 0.870 |
| BAG6 | rs2242656 | 0.787 (0.649-0.954) | 0.297 |  | 0.914 (0.715-1.164) | 0.841 |  | 0.663 (0.493-0.883) | 0.113 |
| BAG6 | rs9380266 | 0.998 (0.833-1.196) | 0.998 |  | 1.002 (0.795-1.262) | 0.985 |  | 1.033 (0.795-1.340) | 0.927 |
| BAG6 | rs1077394 | 0.913 (0.765-1.090) | 0.709 |  | 0.987 (0.787-1.239) | 0.985 |  | 0.898 (0.694-1.160) | 0.819 |
| BAG6 | rs3130048 | 0.968 (0.810-1.157) | 0.901 |  | 0.990 (0.787-1.245) | 0.985 |  | 0.909 (0.703-1.178) | 0.819 |
| BAG6 | rs1077393 | 0.719 (0.594-0.869) | 0.020 |  | 0.814 (0.638-1.040) | 0.654 |  | 0.695 (0.529-0.916) | 0.113 |
| BAG6 | rs1052486 | 0.920 (0.762-1.110) | 0.784 |  | 1.029 (0.807-1.315) | 0.985 |  | 0.832 (0.636-1.090) | 0.637 |
| BAG6 | rs2844463 | 0.838 (0.700-1.002) | 0.420 |  | 0.955 (0.757-1.203) | 0.970 |  | 0.710 (0.545-0.923) | 0.113 |
| CAMKK1 | rs7214723 | 1.099 (0.918-1.315) | 0.709 |  | 0.993 (0.790-1.250) | 0.985 |  | 1.119 (0.862-1.455) | 0.819 |
| CHEK2 | rs2236141 | 1.092 (0.894-1.334) | 0.784 |  | 0.911 (0.697-1.184) | 0.841 |  | 1.351 (1.017-1.790) | 0.278 |
| CHRNA6 | rs16891604 | 0.806 (0.667-0.973) | 0.303 |  | 0.884 (0.695-1.123) | 0.841 |  | 0.665 (0.497-0.882) | 0.113 |
| CHRNA6 | rs9298628 | 1.062 (0.888-1.272) | 0.901 |  | 0.931 (0.737-1.174) | 0.859 |  | 1.141 (0.881-1.478) | 0.803 |
| CHRNB3 | rs16891569 | 0.844 (0.662-1.073) | 0.549 |  | 0.910 (0.665-1.233) | 0.859 |  | 0.788 (0.549-1.116) | 0.637 |
| CHRNB3 | rs4954 | 0.961 (0.785-1.177) | 0.901 |  | 0.807 (0.615-1.053) | 0.654 |  | 1.139 (0.852-1.515) | 0.819 |
| CHRNB3 | rs16891561 | 0.956 (0.797-1.145) | 0.901 |  | 0.925 (0.732-1.166) | 0.841 |  | 1.033 (0.795-1.341) | 0.927 |
| CHRNB3 | rs4236926 | 0.966 (0.806-1.157) | 0.901 |  | 0.908 (0.718-1.145) | 0.841 |  | 1.030 (0.793-1.336) | 0.927 |
| CLPTM1L | rs31489 | 0.966 (0.790-1.181) | 0.901 |  | 0.988 (0.762-1.275) | 0.985 |  | 1.002 (0.746-1.338) | 0.997 |
| CLPTM1L | rs402710 | 0.971 (0.814-1.160) | 0.901 |  | 0.915 (0.728-1.148) | 0.841 |  | 1.010 (0.782-1.306) | 0.975 |
| CRP | rs2808630 | 1.209 (0.996-1.467) | 0.420 |  | 1.152 (0.897-1.475) | 0.841 |  | 1.353 (1.027-1.777) | 0.268 |
| EGFR | rs763317 | 0.974 (0.809-1.171) | 0.901 |  | 0.918 (0.721-1.166) | 0.841 |  | 0.948 (0.724-1.237) | 0.927 |
| EPHX1 | rs1051741 | 1.056 (0.841-1.328) | 0.901 |  | 1.025 (0.760-1.373) | 0.985 |  | 0.967 (0.687-1.344) | 0.935 |
| EPHX1 | rs2292568 | 0.881 (0.719-1.079) | 0.599 |  | 0.949 (0.730-1.227) | 0.970 |  | 0.863 (0.640-1.156) | 0.803 |
| ERCC2 | rs1799793 | 1.155 (0.885-1.509) | 0.708 |  | 1.309 (0.935-1.817) | 0.654 |  | 0.848 (0.557-1.262) | 0.819 |
| ERCC2 | rs13181 | 1.242 (0.966-1.598) | 0.469 |  | 1.140 (0.821-1.571) | 0.841 |  | 1.078 (0.739-1.550) | 0.927 |
| GSTP1 | rs1695 | 0.854 (0.708-1.030) | 0.469 |  | 0.815 (0.638-1.037) | 0.654 |  | 0.914 (0.696-1.197) | 0.870 |
| IL1B | rs12621220 | 1.051 (0.875-1.264) | 0.901 |  | 1.017 (0.804-1.289) | 0.985 |  | 1.122 (0.859-1.471) | 0.819 |
| IL1B | rs1143623 | 1.000 (0.832-1.202) | 0.998 |  | 1.051 (0.830-1.335) | 0.970 |  | 1.001 (0.767-1.309) | 0.997 |
| IL1B | rs16944 | 1.031 (0.848-1.254) | 0.901 |  | 1.091 (0.848-1.409) | 0.841 |  | 0.962 (0.727-1.279) | 0.927 |
| IL1B | rs3136558 | 1.025 (0.853-1.232) | 0.901 |  | 0.955 (0.755-1.209) | 0.970 |  | 1.041 (0.797-1.363) | 0.927 |
| IL1B | rs1143627 | 1.022 (0.837-1.248) | 0.923 |  | 1.113 (0.859-1.450) | 0.841 |  | 0.922 (0.694-1.230) | 0.895 |
| IL1RAP | rs4687163 | 0.964 (0.798-1.166) | 0.901 |  | 0.862 (0.672-1.101) | 0.841 |  | 1.111 (0.844-1.458) | 0.819 |
| MMP12 | rs586701 | 1.183 (0.970-1.444) | 0.469 |  | 1.265 (0.983-1.624) | 0.654 |  | 1.090 (0.812-1.454) | 0.895 |
| MMP2 | rs2285053 | 0.973 (0.812-1.165) | 0.901 |  | 0.973 (0.771-1.225) | 0.985 |  | 1.102 (0.850-1.428) | 0.819 |
| MMP2 | rs243865 | 1.058 (0.851-1.316) | 0.901 |  | 1.246 (0.948-1.632) | 0.654 |  | 0.977 (0.704-1.342) | 0.965 |
| MMP9 | rs2250889 | 0.950 (0.795-1.134) | 0.901 |  | 0.862 (0.685-1.082) | 0.785 |  | 1.034 (0.799-1.338) | 0.927 |
| MTHFR | rs17037396 | 0.912 (0.726-1.144) | 0.784 |  | 0.966 (0.721-1.286) | 0.985 |  | 0.934 (0.667-1.295) | 0.927 |
| MTHFR | rs1801133 | 0.847 (0.698-1.028) | 0.469 |  | 0.905 (0.706-1.165) | 0.841 |  | 0.700 (0.532-0.923) | 0.113 |
| NQO1 | rs1800566 | 0.880 (0.724-1.070) | 0.599 |  | 0.865 (0.675-1.112) | 0.841 |  | 0.864 (0.653-1.148) | 0.803 |
| RBMS3 | rs1530057 | 1.004 (0.798-1.263) | 0.998 |  | 0.994 (0.739-1.328) | 0.985 |  | 0.827 (0.580-1.161) | 0.803 |
| TERT | rs6554759 | 1.020 (0.751-1.385) | 0.959 |  | 0.893 (0.591-1.324) | 0.886 |  | 1.129 (0.719-1.735) | 0.895 |
| TERT | rs2736122 | 0.924 (0.702-1.214) | 0.901 |  | 0.945 (0.662-1.334) | 0.985 |  | 0.937 (0.622-1.385) | 0.927 |
| TERT | rs4635969 | 0.836 (0.666-1.049) | 0.517 |  | 0.876 (0.652-1.169) | 0.841 |  | 0.873 (0.623-1.208) | 0.819 |
| TERT | rs4975605 | 0.900 (0.717-1.127) | 0.782 |  | 0.804 (0.593-1.080) | 0.773 |  | 1.049 (0.756-1.442) | 0.927 |
| TERT | rs2075786 | 0.863 (0.708-1.051) | 0.517 |  | 1.012 (0.788-1.296) | 0.985 |  | 0.766 (0.569-1.024) | 0.384 |
| TERT | rs10069690 | 0.984 (0.812-1.194) | 0.951 |  | 1.134 (0.889-1.444) | 0.841 |  | 0.821 (0.615-1.091) | 0.637 |
| TERT | rs2853676 | 0.976 (0.808-1.178) | 0.901 |  | 0.995 (0.781-1.266) | 0.985 |  | 0.936 (0.709-1.229) | 0.927 |
| TERT | rs2735845 | 1.229 (1.027-1.471) | 0.303 |  | 1.160 (0.922-1.461) | 0.785 |  | 1.289 (0.993-1.678) | 0.365 |
| TERT | rs4246742 | 1.133 (0.948-1.356) | 0.549 |  | 1.209 (0.960-1.525) | 0.654 |  | 1.014 (0.783-1.314) | 0.975 |
| TERT | rs2853668 | 1.206 (1.007-1.445) | 0.420 |  | 1.171 (0.928-1.478) | 0.785 |  | 1.275 (0.981-1.660) | 0.384 |
| TGFBR2 | rs3087465 | 0.923 (0.763-1.116) | 0.784 |  | 0.900 (0.703-1.149) | 0.841 |  | 0.945 (0.717-1.239) | 0.927 |
| TGFBR2 | rs2228048 | 1.037 (0.868-1.238) | 0.901 |  | 1.239 (0.988-1.555) | 0.654 |  | 0.794 (0.610-1.031) | 0.399 |
| TGFBR2 | rs3773658 | 1.144 (0.959-1.365) | 0.517 |  | 1.158 (0.923-1.454) | 0.785 |  | 1.280 (0.990-1.657) | 0.365 |
| TGFBR2 | rs9790292 | 1.150 (0.960-1.378) | 0.517 |  | 1.296 (1.025-1.641) | 0.654 |  | 0.991 (0.764-1.286) | 0.975 |
| TGFBR2 | rs3773663 | 1.198 (0.982-1.461) | 0.469 |  | 1.235 (0.956-1.603) | 0.654 |  | 1.252 (0.937-1.684) | 0.541 |
| TYMS | rs3819102 | 1.403 (1.172-1.680) | 0.014 |  | 1.407 (1.118-1.772) | 0.222 |  | 1.533 (1.181-1.988) | 0.079 |
| XPA | rs1800975 | 1.122 (0.915-1.377) | 0.684 |  | 1.113 (0.857-1.452) | 0.841 |  | 1.196 (0.888-1.623) | 0.787 |
| XRCC6 | rs2267437 | 0.888 (0.736-1.072) | 0.599 |  | 0.906 (0.709-1.153) | 0.841 |  | 0.858 (0.650-1.126) | 0.803 |

^a^ p-values were calculated in in multivariate logistic regression (adjust for sex, age) after false discovery rate (FDR) adjustment for multiple testing

SNP: single nucleotide polymorphism

OR: odds ratio

CI: confidence interval

ADC: lung adenocarcinoma

SCC: lung squamous cell carcinoma
