# Supplementary material for: Prediction of lung cancer risk in Chinese population with genetic‐environment factor using extreme gradient boosting
Source: Cancer Med. 2022 May 2;11(23):4469–78. doi: 10.1002/cam4.4800 (PMC9741969; doi:10.1002/cam4.4800)
Supplement: Supplementary file 5 — Table S5 [file CAM4-11-4469-s004.docx]

**Supplementary Table 5 Association between lung cancer and SNPs in recessive model**

|  |  | Lung cancer | |  | ADC | |  | SCC | |
| --- | --- | --- | --- | --- | --- | --- | --- | --- | --- |
| Gene | SNP | OR (95% CI) ^a^ | P ^a^ |  | OR (95% CI) ^a^ | P ^a^ |  | OR (95% CI) ^a^ | P ^a^ |
| ARHGEF11 | rs868188 | 1.051 (0.847-1.305) | 0.839 |  | 1.326 (1.015-1.727) | 0.361 |  | 0.819 (0.585-1.132) | 0.838 |
| BAG6 | rs3130628 | 1.577 (0.687-3.809) | 0.667 |  | 1.176 (0.316-3.654) | 0.933 |  | 1.664 (0.552-4.669) | 0.864 |
| BAG6 | rs3130047 | 1.463 (0.628-3.562) | 0.710 |  | 1.662 (0.551-4.666) | 0.800 |  | 1.382 (0.418-4.090) | 0.896 |
| BAG6 | rs805298 | 0.705 (0.338-1.424) | 0.667 |  | 0.825 (0.319-1.904) | 0.832 |  | 0.683 (0.194-1.881) | 0.876 |
| BAG6 | rs2077102 | 0.798 (0.429-1.465) | 0.746 |  | 0.625 (0.229-1.453) | 0.800 |  | 0.639 (0.233-1.495) | 0.864 |
| BAG6 | rs2242656 | 0.692 (0.400-1.177) | 0.523 |  | 0.730 (0.349-1.414) | 0.812 |  | 0.763 (0.321-1.614) | 0.876 |
| BAG6 | rs9380266 | 1.077 (0.719-1.614) | 0.878 |  | 0.988 (0.566-1.665) | 0.984 |  | 1.196 (0.687-2.018) | 0.876 |
| BAG6 | rs1077394 | 0.669 (0.497-0.896) | 0.222 |  | 0.628 (0.417-0.922) | 0.350 |  | 0.558 (0.341-0.879) | 0.235 |
| BAG6 | rs3130048 | 1.154 (0.883-1.509) | 0.667 |  | 1.212 (0.858-1.695) | 0.800 |  | 1.150 (0.785-1.662) | 0.876 |
| BAG6 | rs1077393 | 0.810 (0.642-1.021) | 0.405 |  | 0.858 (0.634-1.152) | 0.800 |  | 0.692 (0.480-0.980) | 0.521 |
| BAG6 | rs1052486 | 0.868 (0.692-1.087) | 0.578 |  | 0.908 (0.678-1.208) | 0.817 |  | 0.717 (0.502-1.007) | 0.612 |
| BAG6 | rs2844463 | 0.903 (0.641-1.268) | 0.793 |  | 0.879 (0.555-1.359) | 0.820 |  | 0.844 (0.496-1.384) | 0.876 |
| CAMKK1 | rs7214723 | 0.743 (0.566-0.973) | 0.318 |  | 0.722 (0.498-1.028) | 0.588 |  | 0.829 (0.559-1.205) | 0.864 |
| CHEK2 | rs2236141 | 1.489 (0.837-2.699) | 0.523 |  | 1.008 (0.431-2.185) | 0.984 |  | 2.770 (1.310-5.758) | 0.199 |
| CHRNA6 | rs16891604 | 0.709 (0.448-1.111) | 0.474 |  | 0.728 (0.395-1.273) | 0.800 |  | 0.667 (0.311-1.298) | 0.838 |
| CHRNA6 | rs9298628 | 0.711 (0.472-1.063) | 0.405 |  | 0.514 (0.273-0.906) | 0.350 |  | 0.991 (0.555-1.695) | 0.987 |
| CHRNB3 | rs16891569 | 0.630 (0.129-2.577) | 0.786 |  | - | 0.984 |  | 0.699 (0.036-4.539) | 0.981 |
| CHRNB3 | rs4954 | 0.805 (0.426-1.497) | 0.753 |  | 0.684 (0.268-1.536) | 0.812 |  | 1.160 (0.474-2.585) | 0.981 |
| CHRNB3 | rs16891561 | 0.539 (0.346-0.824) | 0.222 |  | 0.490 (0.260-0.862) | 0.350 |  | 0.680 (0.350-1.229) | 0.838 |
| CHRNB3 | rs4236926 | 0.583 (0.380-0.884) | 0.246 |  | 0.469 (0.244-0.836) | 0.350 |  | 0.704 (0.371-1.254) | 0.838 |
| CLPTM1L | rs31489 | 0.778 (0.420-1.417) | 0.745 |  | 0.749 (0.313-1.613) | 0.817 |  | 0.601 (0.199-1.487) | 0.864 |
| CLPTM1L | rs402710 | 0.977 (0.722-1.320) | 0.923 |  | 0.975 (0.656-1.424) | 0.960 |  | 0.705 (0.422-1.131) | 0.765 |
| CRP | rs2808630 | 0.892 (0.520-1.520) | 0.839 |  | 0.831 (0.393-1.632) | 0.825 |  | 1.023 (0.464-2.083) | 0.987 |
| EGFR | rs763317 | 1.010 (0.652-1.562) | 0.965 |  | 1.279 (0.747-2.137) | 0.807 |  | 0.693 (0.322-1.358) | 0.864 |
| EPHX1 | rs1051741 | 1.901 (0.720-5.540) | 0.577 |  | 1.703 (0.431-6.037) | 0.817 |  | 1.311 (0.271-5.137) | 0.981 |
| EPHX1 | rs2292568 | 0.692 (0.358-1.301) | 0.658 |  | 0.639 (0.252-1.428) | 0.800 |  | 1.045 (0.429-2.304) | 0.987 |
| ERCC2 | rs1799793 | 1.034 (0.243-4.394) | 0.965 |  | 1.865 (0.362-8.583) | 0.817 |  | 0.672 (0.034-4.751) | 0.981 |
| ERCC2 | rs13181 | 3.842 (0.924-25.865) | 0.405 |  | 2.641 (0.312-22.325) | 0.800 |  | 3.667 (0.600-28.125) | 0.765 |
| GSTP1 | rs1695 | 0.660 (0.399-1.073) | 0.405 |  | 0.679 (0.346-1.249) | 0.800 |  | 0.568 (0.242-1.176) | 0.765 |
| IL1B | rs12621220 | 1.062 (0.836-1.349) | 0.839 |  | 1.083 (0.793-1.466) | 0.825 |  | 1.056 (0.746-1.477) | 0.981 |
| IL1B | rs1143623 | 1.053 (0.830-1.334) | 0.839 |  | 1.101 (0.811-1.484) | 0.817 |  | 1.119 (0.793-1.560) | 0.876 |
| IL1B | rs16944 | 1.027 (0.832-1.267) | 0.920 |  | 1.088 (0.831-1.419) | 0.817 |  | 1.026 (0.754-1.386) | 0.987 |
| IL1B | rs3136558 | 0.913 (0.709-1.174) | 0.746 |  | 0.940 (0.674-1.298) | 0.867 |  | 0.963 (0.671-1.364) | 0.987 |
| IL1B | rs1143627 | 1.048 (0.849-1.294) | 0.839 |  | 1.071 (0.814-1.401) | 0.825 |  | 1.052 (0.773-1.420) | 0.981 |
| IL1RAP | rs4687163 | 0.644 (0.384-1.061) | 0.405 |  | 0.570 (0.267-1.107) | 0.595 |  | 0.573 (0.245-1.185) | 0.765 |
| MMP12 | rs586701 | 1.734 (0.938-3.310) | 0.405 |  | 1.706 (0.759-3.696) | 0.789 |  | 2.045 (0.900-4.505) | 0.681 |
| MMP2 | rs2285053 | 0.895 (0.609-1.313) | 0.793 |  | 0.894 (0.534-1.452) | 0.832 |  | 0.951 (0.540-1.608) | 0.987 |
| MMP2 | rs243865 | 1.394 (0.609-3.287) | 0.746 |  | 1.342 (0.450-3.671) | 0.820 |  | 2.312 (0.745-6.713) | 0.765 |
| MMP9 | rs2250889 | 0.753 (0.520-1.084) | 0.474 |  | 0.581 (0.336-0.959) | 0.361 |  | 0.855 (0.483-1.445) | 0.896 |
| MTHFR | rs17037396 | 2.396 (1.017-6.262) | 0.405 |  | 1.907 (0.606-5.822) | 0.800 |  | 2.672 (0.826-8.488) | 0.694 |
| MTHFR | rs1801133 | 0.847 (0.678-1.056) | 0.474 |  | 0.850 (0.636-1.130) | 0.800 |  | 0.891 (0.643-1.223) | 0.876 |
| NQO1 | rs1800566 | 1.023 (0.827-1.266) | 0.920 |  | 1.198 (0.918-1.557) | 0.789 |  | 0.985 (0.716-1.343) | 0.987 |
| RBMS3 | rs1530057 | 2.656 (1.074-7.480) | 0.386 |  | 2.511 (0.822-7.907) | 0.595 |  | 1.695 (0.343-6.878) | 0.876 |
| TERT | rs6554759 | 1.586 (0.451-6.226) | 0.746 |  | 1.705 (0.332-7.840) | 0.817 |  | 0.9559 (0.048-6.820) | 0.987 |
| TERT | rs2736122 | 0.325 (0.016-2.548) | 0.667 |  | - | 0.984 |  | 0.904 (0.045-7.133) | 0.987 |
| TERT | rs4635969 | 0.909 (0.340-2.389) | 0.920 |  | 1.091 (0.293-3.394) | 0.960 |  | - | 0.987 |
| TERT | rs4975605 | 1.034 (0.440-2.426) | 0.965 |  | 0.623 (0.140-2.023) | 0.817 |  | 0.989 (0.218-3.319) | 0.987 |
| TERT | rs2075786 | 0.496 (0.259-0.911) | 0.318 |  | 0.608 (0.269-1.246) | 0.800 |  | 0.527 (0.177-1.278) | 0.793 |
| TERT | rs10069690 | 1.090 (0.661-1.803) | 0.879 |  | 1.252 (0.676-2.253) | 0.817 |  | 1.071 (0.466-2.248) | 0.987 |
| TERT | rs2853676 | 0.776 (0.463-1.285) | 0.667 |  | 0.548 (0.245-1.103) | 0.595 |  | 1.345 (0.696-2.488) | 0.876 |
| TERT | rs2735845 | 1.202 (0.925-1.564) | 0.523 |  | 1.046 (0.738-1.467) | 0.933 |  | 1.576 (1.099-2.239) | 0.235 |
| TERT | rs4246742 | 1.250 (0.960-1.631) | 0.405 |  | 1.442 (1.038-1.991) | 0.350 |  | 1.104 (0.738-1.625) | 0.947 |
| TERT | rs2853668 | 1.156 (0.855-1.564) | 0.667 |  | 1.155 (0.779-1.689) | 0.817 |  | 1.205 (0.777-1.832) | 0.876 |
| TGFBR2 | rs3087465 | 1.278 (0.795-2.071) | 0.667 |  | 1.058 (0.549-1.950) | 0.960 |  | 1.239 (0.596-2.429) | 0.896 |
| TGFBR2 | rs2228048 | 0.887 (0.641-1.225) | 0.746 |  | 1.047 (0.699-1.544) | 0.945 |  | 0.813 (0.489-1.304) | 0.876 |
| TGFBR2 | rs3773658 | 1.026 (0.752-1.400) | 0.923 |  | 0.911 (0.594-1.367) | 0.832 |  | 0.974 (0.613-1.508) | 0.987 |
| TGFBR2 | rs9790292 | 1.124 (0.879-1.438) | 0.667 |  | 1.301 (0.958-1.756) | 0.595 |  | 0.860 (0.582-1.246) | 0.876 |
| TGFBR2 | rs3773663 | 1.022 (0.831-1.257) | 0.920 |  | 1.018 (0.779-1.324) | 0.960 |  | 0.938 (0.689-1.268) | 0.981 |
| TYMS | rs3819102 | 1.537 (1.071-2.221) | 0.314 |  | 1.178 (0.718-1.890) | 0.817 |  | 2.254 (1.395-3.607) | 0.047 |
| XPA | rs1800975 | 0.971 (0.791-1.192) | 0.911 |  | 1.076 (0.829-1.392) | 0.820 |  | 0.811 (0.593-1.099) | 0.793 |
| XRCC6 | rs2267437 | 0.890 (0.595-1.328) | 0.793 |  | 0.702 (0.390-1.203) | 0.800 |  | 1.039 (0.577-1.792) | 0.987 |

^a^ p-values were calculated in in multivariate logistic regression (adjust for sex, age) after false discovery rate (FDR) adjustment for multiple testing

SNP: single nucleotide polymorphism

OR: odds ratio

CI: confidence interval

ADC: lung adenocarcinoma

SCC: lung squamous cell carcinoma
