# Supplementary material for: Prediction of lung cancer risk in Chinese population with genetic‐environment factor using extreme gradient boosting
Source: Cancer Med. 2022 May 2;11(23):4469–78. doi: 10.1002/cam4.4800 (PMC9741969; doi:10.1002/cam4.4800)
Supplement: Supplementary file 6 — Table S6 [file CAM4-11-4469-s007.docx]

**Supplementary Table 6 Comparison of AUC of lung cancer model with SNPs in random forest model and xgboost model.**

| Dataset | random forest | xgboost |
| --- | --- | --- |
| Lung cancer | 0.707 | 0.759 |
| ADC | 0.592 | 0.699 |
| SCC | 0.799 | 0.816 |
| Male | 0.764 | 0.791 |
| Female | 0.543 | 0.614 |
| Young | 0.639 | 0.718 |
| Elder | 0.725 | 0.761 |
| Nonsmoker | 0.655 | 0.667 |
| Smoker | 0.733 | 0.785 |
| Without family history | 0.642 | 0.734 |
| With family history | 0.764 | 0.790 |
