# Supplementary material for: Prediction of lung cancer risk in Chinese population with genetic‐environment factor using extreme gradient boosting
Source: Cancer Med. 2022 May 2;11(23):4469–78. doi: 10.1002/cam4.4800 (PMC9741969; doi:10.1002/cam4.4800)
Supplement: Supplementary file 7 — Table S7 [file CAM4-11-4469-s006.docx]

**Table2 Association between lung cancer and SNPs in additive model**

|  |  | Without family history | |  | With family history | |
| --- | --- | --- | --- | --- | --- | --- |
| Gene | SNP | OR (95% CI) ^a^ | P ^a^ |  | OR (95% CI) ^a^ | P ^a^ |
| ARHGEF11 | rs868188 | 1.042 (0.891-1.220) | 0.806 |  | 1.122 (0.886-1.422) | 0.691 |
| BAG6 | rs1052486 | 0.916 (0.785-1.068) | 0.806 |  | 0.961 (0.768-1.202) | 0.879 |
| BAG6 | rs1077393 | 0.848 (0.726-0.990) | 0.449 |  | 0.741 (0.588-0.931) | 0.216 |
| BAG6 | rs1077394 | 0.846 (0.717-0.998) | 0.449 |  | 0.945 (0.749-1.194) | 0.879 |
| BAG6 | rs2077102 | 0.921 (0.741-1.143) | 0.806 |  | 1.342 (0.975-1.853) | 0.519 |
| BAG6 | rs2242656 | 0.886 (0.719-1.089) | 0.806 |  | 0.653 (0.483-0.878) | 0.216 |
| BAG6 | rs2844463 | 0.918 (0.773-1.090) | 0.806 |  | 0.810 (0.622-1.052) | 0.519 |
| BAG6 | rs3130047 | 1.102 (0.873-1.394) | 0.806 |  | 0.905 (0.623-1.312) | 0.847 |
| BAG6 | rs3130048 | 1.089 (0.928-1.277) | 0.806 |  | 0.887 (0.701-1.122) | 0.691 |
| BAG6 | rs3130628 | 1.005 (0.787-1.282) | 0.981 |  | 0.939 (0.654-1.349) | 0.879 |
| BAG6 | rs805298 | 1.007 (0.796-1.273) | 0.981 |  | 0.651 (0.467-0.903) | 0.216 |
| BAG6 | rs9380266 | 1.050 (0.871-1.266) | 0.806 |  | 0.927 (0.714-1.202) | 0.824 |
| CAMKK1 | rs7214723 | 0.902 (0.767-1.059) | 0.806 |  | 1.197 (0.947-1.517) | 0.519 |
| CHEK2 | rs2236141 | 1.262 (1.019-1.565) | 0.449 |  | 0.896 (0.651-1.231) | 0.824 |
| CHRNA6 | rs16891604 | 0.855 (0.703-1.038) | 0.649 |  | 0.805 (0.604-1.070) | 0.519 |
| CHRNA6 | rs9298628 | 1.002 (0.836-1.201) | 0.981 |  | 0.995 (0.756-1.309) | 0.969 |
| CHRNB3 | rs16891561 | 0.916 (0.763-1.098) | 0.806 |  | 0.843 (0.633-1.119) | 0.620 |
| CHRNB3 | rs16891569 | 0.857 (0.648-1.132) | 0.806 |  | 0.806 (0.520-1.246) | 0.691 |
| CHRNB3 | rs4236926 | 0.927 (0.772-1.112) | 0.806 |  | 0.891 (0.674-1.175) | 0.787 |
| CHRNB3 | rs4954 | 0.955 (0.767-1.188) | 0.826 |  | 0.964 (0.693-1.341) | 0.902 |
| CLPTM1L | rs31489 | 0.960 (0.772-1.194) | 0.855 |  | 1.008 (0.732-1.388) | 0.969 |
| CLPTM1L | rs402710 | 0.963 (0.818-1.133) | 0.813 |  | 1.055 (0.823-1.353) | 0.879 |
| CRP | rs2808630 | 1.086 (0.881-1.338) | 0.806 |  | 1.261 (0.938-1.701) | 0.519 |
| EGFR | rs763317 | 1.014 (0.838-1.229) | 0.962 |  | 0.952 (0.723-1.252) | 0.879 |
| EPHX1 | rs1051741 | 1.082 (0.829-1.412) | 0.806 |  | 1.191 (0.822-1.729) | 0.702 |
| EPHX1 | rs2292568 | 0.835 (0.667-1.043) | 0.649 |  | 0.899 (0.649-1.243) | 0.824 |
| ERCC2 | rs13181 | 1.339 (0.990-1.815) | 0.449 |  | 1.081 (0.716-1.633) | 0.879 |
| ERCC2 | rs1799793 | 1.090 (0.786-1.513) | 0.806 |  | 1.139 (0.748-1.739) | 0.824 |
| GSTP1 | rs1695 | 0.760 (0.622-0.926) | 0.206 |  | 1.094 (0.818-1.463) | 0.824 |
| IL1B | rs1143623 | 1.053 (0.899-1.234) | 0.806 |  | 0.980 (0.788-1.219) | 0.902 |
| IL1B | rs1143627 | 1.053 (0.900-1.232) | 0.806 |  | 1.036 (0.836-1.284) | 0.879 |
| IL1B | rs12621220 | 1.037 (0.885-1.215) | 0.813 |  | 1.124 (0.900-1.405) | 0.691 |
| IL1B | rs16944 | 1.044 (0.894-1.220) | 0.806 |  | 1.037 (0.839-1.282) | 0.879 |
| IL1B | rs3136558 | 1.064 (0.905-1.250) | 0.806 |  | 0.874 (0.696-1.096) | 0.620 |
| IL1RAP | rs4687163 | 0.992 (0.811-1.214) | 0.981 |  | 0.794 (0.594-1.058) | 0.519 |
| MMP12 | rs586701 | 1.188 (0.958-1.475) | 0.649 |  | 1.251 (0.907-1.735) | 0.570 |
| MMP2 | rs2285053 | 0.957 (0.793-1.154) | 0.813 |  | 0.978 (0.763-1.253) | 0.902 |
| MMP2 | rs243865 | 1.116 (0.875-1.424) | 0.806 |  | 0.875 (0.603-1.269) | 0.824 |
| MMP9 | rs2250889 | 0.880 (0.735-1.053) | 0.774 |  | 0.920 (0.714-1.185) | 0.824 |
| MTHFR | rs17037396 | 0.956 (0.737-1.240) | 0.860 |  | 0.979 (0.685-1.399) | 0.936 |
| MTHFR | rs1801133 | 0.922 (0.789-1.076) | 0.806 |  | 0.834 (0.665-1.045) | 0.519 |
| NQO1 | rs1800566 | 1.077 (0.925-1.255) | 0.806 |  | 0.790 (0.630-0.989) | 0.410 |
| RBMS3 | rs1530057 | 1.281 (0.991-1.660) | 0.449 |  | 0.688 (0.464-1.012) | 0.518 |
| TERT | rs10069690 | 0.919 (0.752-1.122) | 0.806 |  | 1.175 (0.860-1.611) | 0.691 |
| TERT | rs2075786 | 0.849 (0.682-1.054) | 0.707 |  | 0.767 (0.562-1.041) | 0.519 |
| TERT | rs2735845 | 1.179 (1.006-1.383) | 0.449 |  | 1.132 (0.897-1.430) | 0.691 |
| TERT | rs2736122 | 1.006 (0.724-1.396) | 0.981 |  | 0.738 (0.456-1.188) | 0.619 |
| TERT | rs2853668 | 1.063 (0.899-1.258) | 0.806 |  | 1.325 (1.033-1.703) | 0.332 |
| TERT | rs2853676 | 0.898 (0.738-1.092) | 0.806 |  | 1.097 (0.805-1.495) | 0.824 |
| TERT | rs4246742 | 1.081 (0.923-1.266) | 0.806 |  | 1.240 (0.976-1.580) | 0.519 |
| TERT | rs4635969 | 0.848 (0.650-1.102) | 0.806 |  | 0.869 (0.598-1.259) | 0.824 |
| TERT | rs4975605 | 1.020 (0.789-1.318) | 0.962 |  | 0.654 (0.449-0.947) | 0.332 |
| TERT | rs6554759 | 1.094 (0.777-1.540) | 0.806 |  | 1.076 (0.634-1.821) | 0.902 |
| TGFBR2 | rs2228048 | 0.979 (0.826-1.160) | 0.908 |  | 0.969 (0.756-1.241) | 0.902 |
| TGFBR2 | rs3087465 | 0.948 (0.779-1.153) | 0.806 |  | 1.052 (0.782-1.416) | 0.879 |
| TGFBR2 | rs3773658 | 1.072 (0.904-1.272) | 0.806 |  | 1.093 (0.863-1.386) | 0.824 |
| TGFBR2 | rs3773663 | 1.050 (0.902-1.222) | 0.806 |  | 1.165 (0.933-1.457) | 0.570 |
| TGFBR2 | rs9790292 | 1.057 (0.904-1.235) | 0.806 |  | 1.145 (0.914-1.436) | 0.620 |
| TYMS | rs3819102 | 1.434 (1.200-1.715) | 0.005 |  | 1.192 (0.919-1.549) | 0.570 |
| XPA | rs1800975 | 0.940 (0.806-1.095) | 0.806 |  | 0.978 (0.779-1.227) | 0.902 |
| XRCC6 | rs2267437 | 0.970 (0.803-1.170) | 0.860 |  | 0.832 (0.634-1.091) | 0.570 |

^a^ p-values were calculated in in multivariate logistic regression (adjust for gender, age) after False Discovery Rate (FDR) adjustment for multiple testing

SNP: single nucleotide polymorphism

OR: odds ratio

CI: confidence interval

ADC: lung adenocarcinoma

SCC: lung squamous cell carcinoma
